# Selectivity in Buttress Drumming Tree Properties Among Chimpanzees (*Pan troglodytes schweinfurthii*) of the Waibira Community in Budongo Forest, Uganda

**DOI:** 10.1002/ajp.23712

**Published:** 2024-12-25

**Authors:** Wytse Wilhelm, Vesta Eleuteri, Kathelijne Koops, Maegan Fitzgerald, Klaus Zuberbühler, Catherine Hobaiter

**Affiliations:** ^1^ Wild Minds Lab, School of Psychology and Neuroscience University of St Andrews Fife United Kingdom; ^2^ Pathways to Language Group, Institute for Archaeological Sciences University of Tübingen Tübingen Germany; ^3^ Department of Behavioral and Cognitive Biology University of Vienna Vienna Austria; ^4^ Ape Behaviour & Ecology Group, Department of Evolutionary Anthropology University of Zurich Zurich Switzerland; ^5^ Budongo Conservation Field Station Masindi Uganda; ^6^ Institute of Biology University of Neuchâtel Neuchâtel Switzerland

**Keywords:** behavioral selectivity, bioacoustics, eastern chimpanzees, long‐distance communication

## Abstract

Wild chimpanzees drum on tree buttresses during dominance displays and travel, generating low‐frequency sounds that are audible over distances of more than 1 km. Western chimpanzees (*Pan troglodytes verus*) in the Nimba Mountains of Guinea selectively choose trees and buttresses when drumming, potentially based on their resonant properties, suggesting that these chimpanzees are optimizing their drumming signals. We investigated whether male eastern chimpanzees (*Pan troglodytes schweinfurthii*) from the Waibira community in the Budongo Forest, Uganda, also show preferences in tree and buttress choice, exploring whether selectivity is a species‐wide feature. We tested chimpanzee preferences for tree species and diameter, number of buttresses, and buttress area and width, by comparing trees and buttresses used in drumming bouts with nearby unused trees and buttresses. Waibira chimpanzees drummed preferentially on two tree species: the tropical hardwood *Cynometra alexandrii* and the softwood *Chrysophyllum albidum*. Chimpanzees selected trees with a larger diameter over nearby trees with a smaller diameter, and buttresses were more likely to be used for drumming if they had a larger area or larger width. These results suggest that chimpanzees in the Waibira community select trees and buttresses based on physical properties, most likely related to acoustically relevant characteristics. These findings support the argument that buttress drumming is a goal‐directed behavior and contributes to our understanding of chimpanzees' use and optimization of their long‐distance acoustic signals.

## Summary

- Waibira chimpanzees in the Budongo Forest, Uganda, drummed preferentially on the buttress roots of two tree species: the tropical hardwood *Cynometra alexandrii* and the softwood *Chrysophyllum albidum*.
- Waibira chimpanzees selected trees with a larger diameter over nearby trees with a smaller diameter, and buttresses were more likely to be used for drumming if they had a larger area or larger width.
- Chimpanzees across Africa select trees and buttresses based on physical characteristics likely to optimize acoustic resonance.

## Abstract (French)

Les chimpanzés sauvages tambourinent sur les contreforts des arbres lors des démonstrations de dominance et des déplacements, générant des sons de basse fréquence audibles sur des distances de plus d'un kilomètre. Les chimpanzés de l'Ouest (*Pan troglodytes verus*) des Monts Nimba en Guinée choisissent sélectivement les arbres et les contreforts lorsqu'ils tambourinent, potentiellement en fonction de leurs propriétés de résonance (Fitzgerald et al., 2022), ce qui suggère que ces chimpanzés optimisent leur tambourinage. Nous avons cherché à savoir si les chimpanzés mâles de l'Est (*Pan troglodytes schweinfurthii*) de la communauté Waibira, de la forêt de Budongo, en Ouganda, montraient également des préférences dans le choix des arbres et des contreforts, afin d'explorer si la sélectivité est une caractéristique de l'espèce en general. Nous avons testé les préférences des chimpanzés pour les espèces d'arbres et leur diamètre, le nombre de contreforts, et la surface et la largeur des contreforts, en comparant les arbres et les contreforts utilisés lors des séances de tambourinage avec les arbres non utilisés à proximité et les contreforts. Les chimpanzés de Waibira tambourinent de préférence sur deux espèces d'arbres: le feuillu tropical *Cynometra alexandrii* et le résineux *Chrysophyllum albidum*. Les chimpanzés ont choisi des arbres de plus grand diamètre plutôt que des arbres voisins de plus petit diamètre, et les contreforts étaient plus susceptibles d'être utilisés pour le tambourinage s'ils avaient une plus grande surface ou une plus grande largeur. Ces résultats suggèrent que les chimpanzés de la communauté de Waibira sélectionnent les arbres et les contreforts en fonction de leurs propriétés physiques, très probablement liées à des caractéristiques acoustiques pertinentes. Ces résultats contribuent à notre compréhension de l'utilisation et de l'optimisation par les chimpanzés de leurs signaux acoustiques à longue distance.

## 1 Introduction

Auditory communication exploiting tools or substrates appears in a wide variety of animal species (e.g., spiders: Hebets et al. 2008; mammals: Randall 2001; birds: Slabbekoorn, Ellers, and Smith 2002). One purpose is to modulate and increase call efficacy. For example, tree‐hole frogs (*Metaphrynella sundana*) adapt the frequency of their vocalizations to the resonance features of tree‐holes, making their calls deeper and louder, thereby attracting more females (Lardner and bin Lakim 2002). Similarly, Mientien tree frogs (*Kurixalus idiootocus*) prefer to call from inside concrete drainages that enhance the amplitude and length of their calls (Tan et al. 2014). Tree crickets (*Oecanthus henryi*) make their own acoustic baffles by fashioning holes in leaves that augment the loudness of their mate‐attraction calls and select larger leaves for increased efficacy (Mhatre et al. 2017). Bornean orangutans (*Pongo pygmaeus wurmbii*) lower the frequency of their kiss‐squeak alarm calls with leaves when they are in more dangerous situations, which may function to deceptively convey a larger body size (Hardus et al. 2009).

Drumming signals are particularly common nonvocal signals, employed by diverse species for purposes ranging from territorial defence to coordination of mating interactions, suggesting that percussive behavior evolved independently in multiple lineages and for different purposes (Randall 2001). For example, mole rats (*Bathyergidea* family) drum on the ground with their hind paws to warn conspecifics of their presence (Jarvis and Bennett 1991), while great gerbils (*Rhombomys opimus*) employ the same behavior to warn conspecifics of nearby predators (Randall, Rogovin, and Shier 2000). Male palm cockatoos (*Probosciger aterrimus*) employ tools to drum, fashioning modified seed pods and sticks for use during their mating displays (Heinsohn et al. 2017).

An especially impressive form of drumming is performed by wild chimpanzees (*Pan troglodytes*), most commonly by pounding their hands and feet on the buttress roots of large trees (Goodall 1965, 1986; Reynolds and Reynolds 1965). Drumming, along with their pant‐hoot vocalizations, provides chimpanzees with an effective and flexible long‐distance signal (Goodall 1986), traveling over a kilometer in dense forest habitats (Boesch 1991). However, while pant‐hooting is the most commonly studied signal in chimpanzees (e.g., Arcadi 1996; Desai et al. 2022; Fedurek, Donnellan, and Slocombe 2014; Fedurek et al. 2013; Fedurek, Schel, and Slocombe 2013; Soldati et al. 2022), drumming has been comparably less well‐studied, with a handful of studies on social and contextual variables (e.g., Boesch 1991; Arcadi, Robert, and Boesch 1998; Babiszewska et al. 2015; Eleuteri et al. 2022), acoustic features (Arcadi, Robert, and Boesch 1998, Arcadi and Wallauer 2013, Babiszewska et al. 2015, Eleuteri et al. 2022), and tree and buttress selection (Fitzgerald et al. 2022).

Originally thought to function as a dominance signal, similar to chest‐beating in gorillas (Goodall 1968, 1986; Marler 1976; Nishida et al. 1999), our current understanding of chimpanzee buttress drumming suggests it is used for both near‐by and long‐distance signaling, with potentially distinct functions. Chimpanzees live in fission‐fusion communities, with individuals often traveling in different small parties for periods of time, depending on available resources and mating opportunities (e.g., Anderson et al. 2002; Lehmann and Boesch 2004; Newton‐Fisher, Reynolds, and Plumptre 2000). Long‐distance drumming appears to be related to this social organization, allowing individuals to communicate with distant group members to coordinate travel (Arcadi, Robert, and Boesch 1998; Babiszewska et al. 2015; Eleuteri et al. 2022). The majority of travel pant‐hoots are accompanied by drumming, which—in this context—encodes information on individual identity (Babiszewska et al. 2015; Eleuteri et al. 2022). In addition, while the composition of the nearby audience does not impact the use of drumming (Babiszewska et al. 2015), the size of the party does. Drumming is less common in larger parties, probably because when a greater proportion of potential recipients is already present, there is less need for chimpanzees to advertise their location and identity (Eleuteri et al. 2022). Interestingly, drumming during dominance displays does not encode individual identity and is also less likely to be accompanied by long‐distance pant‐hoot calls, which suggests a dual function of buttress drumming (Eleuteri et al. 2022).

Chimpanzees show material selectivity in many contexts, including when choosing tools for nut‐cracking (Boesch et al. 2017; Carvalho et al. 2009; Sirianni, Mundry, and Boesch 2015), ant‐dipping (Koops et al. 2014; Nishida 1973), and termite fishing (Almeida‐Warren et al. 2017; Sanz and Morgan 2007). Similarly, they show material preferences when building sleeping‐platforms or “nests,” choosing larger trees and firmer and more stable branches (Hakizimana et al. 2015; Koops et al. 2012; Samson and Hunt 2014). In buttress drumming, Fitzgerald et al. (2022) found that Western chimpanzees (*P. troglodytes verus*) in the Seringbara region of the Nimba Mountains (Guinea) prefer to drum on trees with a larger number of buttresses and with larger and thinner buttresses, two features that likely impact resonance. In human musical instruments, including drums, changes to surface area and thickness are the primary means of varying resonance (Yamaha Corporation 2020). Similar patterns have been found in accumulative stone throwing in which chimpanzees throw stones against trees (Kühl et al. 2016) and show a preference for species with greater resonance (Kalan et al. 2019).

In addition to buttress size and thickness, wood density could also impact the loudness, pitch (i.e., frequency), and tone (i.e., combination of overtone frequencies) of drumming, as it determines the speed with which sound can travel through the material (Wegst 2006). Loudness directly increases the distance sound travels, but so can pitch and tone, as sounds with lower frequency travel further in areas with dense growth (Marten and Marler 1977). The physical properties of the drumming tree could impact drumming production in other ways as well. Chimpanzees frequently drum by holding the edge of the buttress and kicking against it with their feet (Goodall 1986), suggesting that buttress shape and structure could impact control over the volume, length, or patterning of their drumming bout.

Investigating preferences across subspecies can indicate to what extent preferences are shared across populations, subspecies, and/or habitats. Here, we build on work by Fitzgerald et al. (2022), showing a preference for larger trees and thinner buttresses among Western chimpanzees, adding an eastern population. We investigate whether chimpanzees of the Waibira community in the Budongo Forest Reserve, Uganda, showed selectivity when choosing trees and buttresses for drumming. Specifically, we investigated whether certain tree species, tree size, wood density, or buttress structure were preferred, all of which are likely to impact the amplitude, frequency, harmonic structure, and rhythm of drumming (Wegst 2006). Preference in any of these features could indicate that chimpanzees consider the structural and acoustic characteristics of substrates when performing their drumming signals.

## 2 Methods

### 2.1 Ethical Statement

All data collection followed the American Society of Primatologists' principles for the ethical treatment of nonhuman primates and the Code of Best Practices for Field Primatology by the International Primatological Society. The Uganda National Council for Science and Technology, the Ugandan Wildlife Authority, and the Budongo Conservation Field Station gave permission for the research. Ethical approval was granted by the University of St. Andrews Animal Welfare and Ethics Committee.

### 2.2 Study Site and Population

We collected data on adult male chimpanzees in the Waibira community of eastern chimpanzees at the Budongo Conservation Field Station in the Budongo Central Forest Reserve, Uganda (Reynolds, 2005). We focus on adult males both to provide a more direct comparison with previous work (Fitzgerald et al. 2022; Eleuteri et al. 2022), and because while both male and female chimpanzees drum in this population, drumming is far more prevalent in adult male eastern chimpanzees (Babiszewska et al. 2015; Eleuteri et al. 2022). The community's home range lies in the Budongo Central Forest Reserve (1°37′–2°03′ N, 31°22′–31°46′ E) in the Western Rift Valley in western Uganda. The reserve covers an area of 793 km^2^, of which 482 km^2^ is covered by medium altitude semi‐deciduous forest (Eggeling 1947).

The Waibira community has been followed by researchers since 2011 (Samuni et al. 2014), and most independent individuals (those who travel independently of their mothers, an indication of maturity), and all independent males were fully habituated at the time of data collection. We evaluated the scope for sampling bias in our study using the STRANGE framework (Webster and Rutz 2020). In observational work, the goal of this framework is to declare and discuss potential sources of difference between data sets, for example, in terms of socio‐ecological context or individual life history, that may impact the behavior of interest. The Waibira community is a forest‐bound group surrounded by other communities. It consists of around 120 individuals, making it larger than most chimpanzee communities (typically range 30–70 individuals; Wilson et al. 2014), and it has an additional level of social structure (core‐periphery), beyond the typical fission‐fusion structure (Badihi et al. 2022). Despite its large community size, the Waibira territory of 11 km^2^ is relatively small (Badihi et al. 2022; Herbinger et al. 2001). This combination of a large, richly structured community and a small territory may influence fission‐fusion dynamics and how these are regulated through long‐distance communication such as buttress drumming.

In this study, we build on previous work by Fitzgerald et al. (2022) on the two Seringbara communities of Western chimpanzees in the Nimba Mountains in southeastern Guinea. There are several key differences between the Seringbara communities and the Waibira community. The Budongo forest territory of the Waibira community is secondary forest growth (Plumptre 1996), while that of the Seringbara communities is primary growth (Koops 2011). As a result, there are larger trees in the Seringbara forest, with larger buttresses, which could contribute to differences in levels of selectivity, as well as differences in drumming characteristics. Chimpanzee density in the Budongo forest is estimated to be ~ 10× higher than in Nimba (Waibira ~10.9 chimpanzees/km^2^; Seringbara ~1.7 chimpanzees/km^2^; Badihi et al. 2022; Koops 2011). Where party size is constrained by external factors, such as food availability, the larger community size and higher density of Waibira chimpanzees may lead to a larger number of parties in a smaller area, with possible consequences for long‐distance communication between parties. Specifically, having to communicate more often, but over shorter distances, could lead to chimpanzees being less selective in their buttress choice.

### 2.3 Drumming Data Collection

In June and July 2023, we collected ad libitum data on drumming behavior while conducting daily focal follows of adult male chimpanzees (Altmann, 1974). Chimpanzees were followed between 0600 and 1800 h, on a four‐on, two‐off working schedule. Drumming bouts were collected from 12 individuals over a period of 30 days, during approximately 125 h of focal following. For video examples of drumming, please see https://tinyurl.com/chimpdrumming.

We selected focal males for daily follows (Table 1) based on their availability in the morning, with preference given to (a) chimpanzees traveling in larger parties with other adult males as this allowed for more ad libitum sampling across males in the party, and (b) males with fewer drumming data already collected. The same focal individual was followed throughout the day. If a focal individual was lost and could not be relocated for over an hour, we interrupted the follow and selected another focal. Focal males were followed for an average of 7.5 h (SD = 4.2, range = 1.5–15.3).

**Table 1:** List of focal individuals included in data collection. Age was calculated at the start of data collection (June 2023). Age represents the estimated age in years. The exact year of birth was unknown for adult individuals born before habituation of the community, so an indication of estimated accuracy in years is included. Individuals aged 12‐years and younger at the time of habituation were assigned an age based on their physical characteristics, with an error of ±1‐year, older individuals were assigned a larger margin of error.

Individual	Code	Year of birth	Age
Alf	ALF	1998 ± 1	25 ± 1
Ardberg	ARD	2005 ± 1	18 ± 1
Ben	BEN	1992 ± 2	31 ± 2
Daudi	DAU	2004 ± 1	19 ± 1
Fiddich	FID	2002 ± 1	21 ± 1
Ila	ILA	1999 ± 1	24 ± 1
Lafroig	LAF	2003 ± 1	20 ± 1
Lahni	LAN	2002 ± 1	21 ± 1
Macallan	MAC	1996 ± 1	27 ± 1
Masariki	MAS	2003 ± 1	20 ± 1
Mugisha	MUG	2003 ± 1	20 ± 1
Sam	SAM	1999 ± 1	24 ± 1

During follows, we collected continuous data on party composition, as well as any pant‐hoot calls and drums produced by or potentially heard by the focal individual (in practice, those that we could hear while the focal was in sight). For each pant‐hoot or drum, we noted (a) who produced it, (b) whether it was a response to another long‐distance call within the last 60 s, and (c) whether it was part of a chorus and the identity of any other known individuals in the chorus. When any adult male in the focal individual's party (including the focal male) drummed, we recorded the bout with Panasonic HC‐V770 camcorder and a Sennheiser MKE 400 microphone (*N* = 61). Following Arcadi and Wallauer (2013), we defined a drumming bout as a series of drumbeats on a single tree (potentially across several buttresses), with further beats produced on a separate tree considered to be a new drumming bout. In addition, where drumming was interrupted by other behaviors, such as feeding or grooming, we consider the production of further beats on the same tree to be a new drumming bout.

At the end of each video, we dictated which individual produced the drum, as well as the behavioral context before and after the drum (Table 2). We determined the behavioral context based on the behavior that lasted for at least 30 s immediately preceding and following the drum with one exception: if the drum was part of a dominance display, we noted the behavioral context both before and after as “Display,” regardless of how long the dominance display lasted. As previous work with this community suggested that chimpanzees discriminated between display drums and non‐display drums in their expression (Eleuteri et al. 2022), we distinguished drums as either “display” (*N* = 22, where the behavioral context either before or after the drum was display) or “other” (*N* = 36, all other behavioral contexts). We also noted whether a drum was combined with a panthoot vocalization (*N*_with_ = 45, *N*_without_ = 8, *N*_unknown_ = 5).

**Table 2:** Behavioral contexts of the signaller. Context was determined based on the behavior in the 30 s immediately preceding and following the drum (adapted from Eleuteri et al. 2022). Categories were treated as mutually exclusive and only behavior that lasted at least 30 s were included.

Context	Description
Travel	Focal individual moved through the forest
Resting	Focal individual was on the ground without engaging in either traveling, feeding, or a display
Feeding	Focal individual was feeding on the ground or was joining a feeding tree
Display	Focal individual displayed with piloerection, gestures, or calls while other individuals were located nearby
Other	Focal individual was engaged in a behavior other than those described above
Unknown	Focal individual was not visible and his behavior was unknown

We marked the location of the drumming tree using a GPS (Garmin GPSMAP 64 s), by making an inconspicuous mark on the buttress(es) used with a pocketknife and by taking a picture of the tree with the used buttress(es) clearly visible. Doing so allowed us to re‐locate the tree to collect detailed ecological data.

### 2.4 Ecological Data Collection

**Figure 1:**
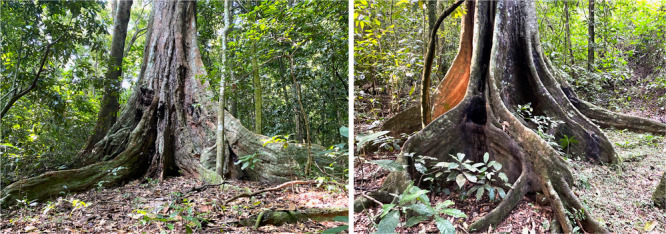
Buttresses on a mature *Cynometra alexandri* and *Celtis mildbraedii*. The largest buttress on the right side of the *Cynometra alexandri* contacts the trunk at approximately 3 m. Whereas the largest buttress on the right side of the smaller *Celtis mildbraedii* contacts the trunk at about 1.2 m. Both species are regularly used for drumming by Waibira chimpanzees.

We collected ecological measurements following the methods established by Fitzgerald et al. (2022). We laid a 20 x 20 m vegetation plot around each unique drumming tree (*N* = 43), which was used to select control trees (*N* = 154) that had not been used in the drumming event. Control trees were required to have a Diameter at Breast Height (DBH; estimated at ~150 cm or where the buttresses met the trunk if this was higher, see Figure 1) of at least 10 cm and have at least one “potential drumming buttress,” defined as any buttress large enough for an adult chimpanzee to hit with the palm of the hand (approximately 200 cm^2^). For each control tree within the plot, we recorded: tree species; diameter at breast height; circumference at breast height (CBH; measured by tape measure at the same height as the diameter at breast height); number of potential drumming buttresses. As trees are not perfect circles, diameter at breast height and circumference at breast height are not perfectly associated, and circumference at breast height is typically a more accurate measure of tree size. However, for *N* = 40 (out of 197) trees, it was impossible to measure the circumference due to the height of the buttresses whereas diameter at breast height could be estimated for all trees. A Spearman's rank correlation test showed that both measures were highly correlated (ρ = 0.94, *p *< 0.001) in this data set, suggesting that our diameter at breast height measures were accurate with respect to the circumference at breast height measure. As a result, we used diameter at breast height as a measure of tree size, as it was available for all trees (see Supporting Information S2 for details of circumference at breast height and diameter at breast height comparison).

For each drumming tree, we recorded the following data on all unique potential drumming buttresses (*N*_drumming buttresses_ = 40, *N*_control buttresses_ = 285):

- Surface area (cm^2^), calculated from the height of the buttress where it meets the trunk of the tree and the length of the base of the buttress, assuming a triangular shape. Although this does not give an exact surface area, it provides a standardized estimate.
- Average width (cm), calculated from average of three points on the buttress: where it met the trunk, where it met the ground, and the midway point. Width was measured using a calliper at a depth of ~10 cm from the edge.

Where a buttress split into two or more buttresses the main buttress was designated based on the continuity of the top of the buttress, as offshoots typically did not reach the full height of the main buttress. We considered any offshoots to be a separate buttress that started at the point of separation and included and measured them if they met the criterion for a potential drumming buttress (minimum area = 200 cm^2^).

Tree species' wood densities were checked against the ICRAF African wood density database (Carsan et al. 2012), which includes measures of density as the ratio of dry weight (at ~12% moisture content after air‐drying) to volume of undried wood.

### 2.5 Statistical Analyses

We performed all statistical tests using base R (v4.2.2; R Core Team 2022), we created plots using ggplot2 (v3.4.0; Wickham 2016) and ggthemes (v4.2.4; Arnold 2021). For full details of all pre‐model assessments of data suitability, see the Supporting Information. All data and code are available in a public repository: https://github.com/Wild-Minds/Chimp-Drumming.

#### 2.5.1 Tree Species

We determined the proportion of times each tree species was used for drumming by dividing the number of drumming bouts on each tree species by the total number of drumming bouts recorded during the study. We then calculated the local availability of drumming tree species by dividing the number of potential drumming trees within the vegetation plots that were of a particular species with the total number of potential drumming trees within the vegetation plots. This calculation provided a separate proportion indicating local availability for each species and a proportion indicating the use of each species for drumming. To assess whether tree species were over‐ or underrepresented among drumming trees compared to their local availability, we performed a binomial test for each species with a Benjamini–Hochberg procedure to correct the *p*‐values for repeated testing (Benjamini and Hochberg 1995). For this analysis, any drumming trees used by multiple individuals were only counted once (*N*_drumming trees_ = 43, *N*_control trees_ = 154).

#### 2.5.2 Tree and Buttress Selection

We investigated whether diameter at breast height and number of buttresses affected the chance of a potential drumming tree to be selected for a drumming bout. We used a binomial GLMM with logit link function (lme4 v1.1‐31; Bates et al. 2015). The dependent variable was whether a tree was used for drumming or not (1/0), with Diameter at Breast Height and Number of Buttresses as predictors. The predictors were normalized with *z*‐transformation to ensure they contributed proportionally to the analysis. We included Context (with the levels “display” and “other”) as a control variable. In addition, we included Drummer ID, Drumming Tree ID and Tree Species as random effects. This method allowed us to include unused trees surrounding a drumming tree as controls, limiting variation in spatial and ecological factors that may play a role in tree selection. For the tree selection model only drumming trees with at least one control tree within the vegetation plot were used, while trees that were used multiple times were included as a separate data point for each drum bout, with the inclusion of Tree ID as a random effect to avoid pseudo‐replication (*N*_drumming trees_ = 18, *N*_control trees_ = 203).

In addition, we investigated whether Buttress Area and Buttress Width influenced the likelihood of a specific buttress on a tree being selected for drumming. We used a binomial GLMM with logit link function (lme4 v1.1‐31; Bates et al. 2015). The dependent variable was whether a buttress was used or not (1/0). The predictors were Buttress Area and Buttress Width. Predictors were *z*‐transformed to ensure they contributed proportionally to the analysis. We included Context as a control variable. We added Drummer ID, Drumming Tree ID, Tree Species, and Drumming bout ID as random effects. This method allowed for trees to function as their own control, with used buttresses on a tree compared with unused buttresses on the same tree. Buttresses that were used multiple times were included as a separate point for each drumming bout (*N*_drumming buttresses_ = 53, *N*_control buttresses_ = 380), with the inclusion of Drum ID and Drumming Tree ID as random effects to control for pseudo‐replication.

We tested models with interaction terms between the predictor variables and the control variables; however, as no interaction terms were significant, we report the results for the models with the predictors as separate main effects (for the interaction models, see Supporting Information S4).

For both models, we used a chi‐squared analysis of variance to compare the fit of the full model with that of a null model, including only the random effect and control variables. We then used a Type II Wald chi‐square test to investigate the significance of the individual predictors (Car v3.1‐1; Fox and Weisberg 2019) and calculated the odds ratio for each significant predictor by exponentiating the estimated coefficients (MuMIn v1.47.5; Barton 2023). To determine the amount of variation in the data explained by the fixed effects, we calculated the conditional *R* ^2^ value (MuMIn v1.47.5; Barton 2023). Then, we used VIFs to test for collinearity between the predictors (Car v3.1‐1; Fox and Weisberg 2019).

In addition to our analyses, we ran a set of replication models with identical structures to those in Fitzgerald and colleagues (2022) to provide a direct comparison. These models differed from our main models by not including Context or all of the random effects, such as individual Drummer ID, which may influence tree selectivity. We found some differences in the significant predictors between our main models and the replication models (for full details, see Supporting Information S5).

Because of the sampling effects of our method, which required us to include all suitable control trees within a fixed area around a single drumming tree within a dense forest, we had a substantial difference between the control and drumming tree (or buttress) sample size. GLMMs are relatively robust to imbalanced samples, but to assess the possible effects of this imbalance we checked variation in the standard deviation of the samples. In the drumming tree model the standard deviation for Diameter at Breast Height (SD_drumming trees_ = 25.7, SD_control trees_ = 23.0) and for Number of Buttresses (SD_drumming trees_ = 2.2, SD_control trees_ = 3.0) were quite similar, but variation was a little larger in the number of buttresses on control trees and we interpret the differences we find with caution in the discussion. In the buttress models, the variation was again fairly similar and was smaller in the (larger) control sample than in the test sample for both Area (SD_drumming buttress_ = 3.1, SD_control trees_ = 1.9) and Width (SD_drumming buttress_ = 2.7, SD_control buttress_ = 1.8)—reducing the chances that differences in sample size would lead to a spuriously significant result.

## 3 Results

We recorded 58 drumming bouts on 43 unique drumming trees, with a total of 40 unique drumming buttresses. Of the total drumming bouts, 22 were display drums, and 36 were from a non‐display context. Drumming bouts were collected from twelve adult male chimpanzees (drums per individual: M = 4.8, SD = 3.5, range = 1–12). We excluded two drumming bouts by subadult males, and for three bouts where we were unable to relocate the drumming tree. Approximately half of the drumming bouts were performed by the focal individual (*N* = 32), with the other half being performed by other adult males in the focal individual's party. On average, focal individuals drummed once every 2 h (*M* = 0.51 drums/hour, SD = 0.51, range = 0.00–1.59). 28% (*N* = 9) of drums by focal individuals were in response to another individual's long‐distance pant‐hoot, and 31% (*N* = 10) either initiated or joined a pant‐hoot or drum chorus.

### 3.1 Tree Species Selection

**Figure 2:**
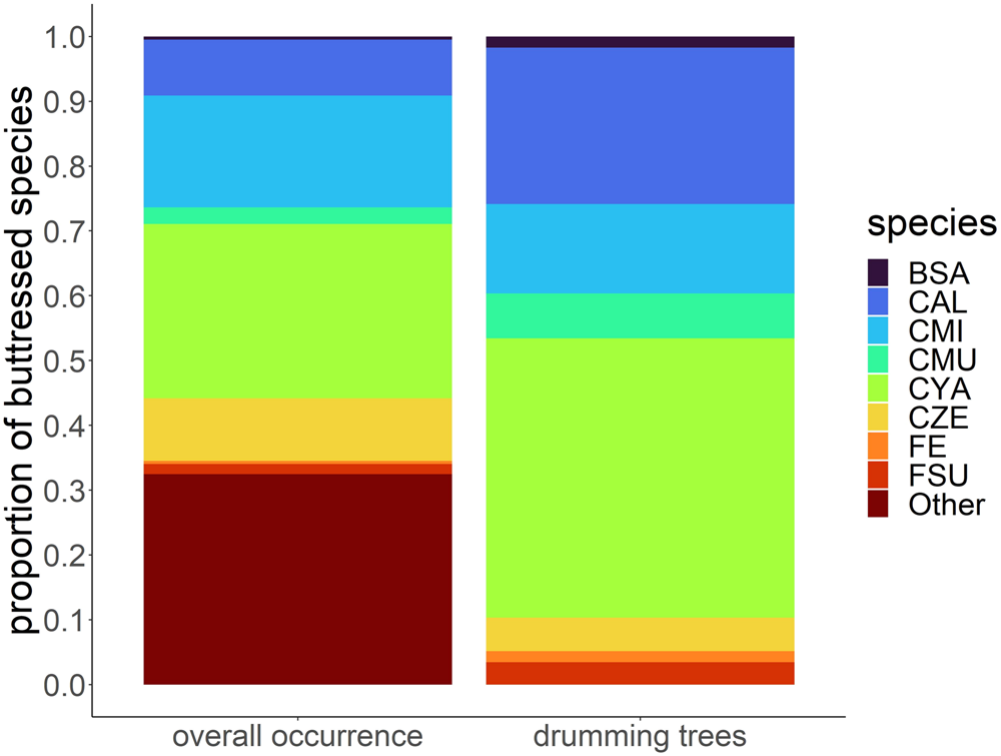
Proportion of buttressed tree species in the territory as a whole and among the drumming trees. On the left is the proportion of each species in the overall vegetation plots, with non‐drumming species grouped under “other.” The largest proportion of an individual species in the territory as a whole is CYA, followed by CMI. Among the drumming trees, the largest proportion is also CYA, followed by CAL. Drumming tree species: *Bersama abyssinica* (BSA); *Chrysophyllum albidum* (CAL); *Celtis mildbraedii* (CMI); *Chrysophyllum muerense* (CMU); *Cynometra alexandrii* (CYA); *Celtis zenkeri* (CZE); *Ficus exasperata* (FA); *Ficus sur* (FSU). Species could not be determined for one tree; however, it was confirmed not to be any of the known species.

We identified 32 different buttressed tree species in the vegetation plots, eight of these were used for drumming (i.e., “drumming trees”; drums per species: M = 7.3, SD = 8.8, range = 1–25; Figure 2). For this analysis drumming trees that were used by multiple individuals were only counted once (*N*_drumming trees_ = 43, *N*_control trees_ = 154).

Two species were overrepresented among drumming trees as compared to their incidence in the territory: *Chrysophyllum albidum* (prop_expected_ = 0.094, prop_drumming_ = 0.241 (95% CI = 0.139–0.372), *p* = 0.006) and *Cynometra alexandrii* (prop_expected_ = 0.244, prop_drumming_ = 0.431 (95% CI = 0.302–0.568), *p* = 0.003). The other species used for drumming were all represented as frequently as expected by chance (Table 3).

**Table 3:** Results of the binomial tests for each drumming tree species. The number of successes is the number of times the species was used for drumming in the 58 drumming bouts (*N* trials). Therefore, the probability of success is the proportion of drumming trees that are that species. The *p*‐values were corrected using the Benjamin*–*Hochsberg procedure for multiple testing. Drumming tree species*: Bersama abyssinica* (BSA), *Chrysophyllum albidum* (CAL), *Celtis mildbraedii* (CMI), *Chrysophyllum muerense* (CMU), *Cynometra alexandrii* (CYA), *Celtis zenkeri* (CZE), *Ficus exasperata* (FA), *Ficus sur* (FSU).

	*N* success	*N* trials	Probability	2.5% CI	97.5% CI	Corrected *p*
BSA	1	58	0.017	0.000	0.092	0.341
CAL	14	58	0.241	0.139	0.372	0.003
CMI	8	58	0.138	0.062	0.254	0.603
CMU	4	58	0.069	0.019	0.167	0.149
CYA	25	58	0.431	0.302	0.568	0.030
CZE	3	58	0.052	0.012	0.144	0.423
FE	1	58	0.017	0.000	0.092	0.341
FSU	2	58	0.035	0.004	0.119	0.341

Of the 32 buttressed tree species identified in our study, 25 had a known density, with an average density of 0.54 g/cm^3^ (SD = 0.15, range = 0.22–0.82). Of the preferred drumming species, *C. alexandrii* has a density of 0.82 g/cm^3^, the highest of the identified species, while *C. albidum* has a density of 0.64 g/cm^3^.

### 3.2 Individual Tree Selection

**Figure 3:**
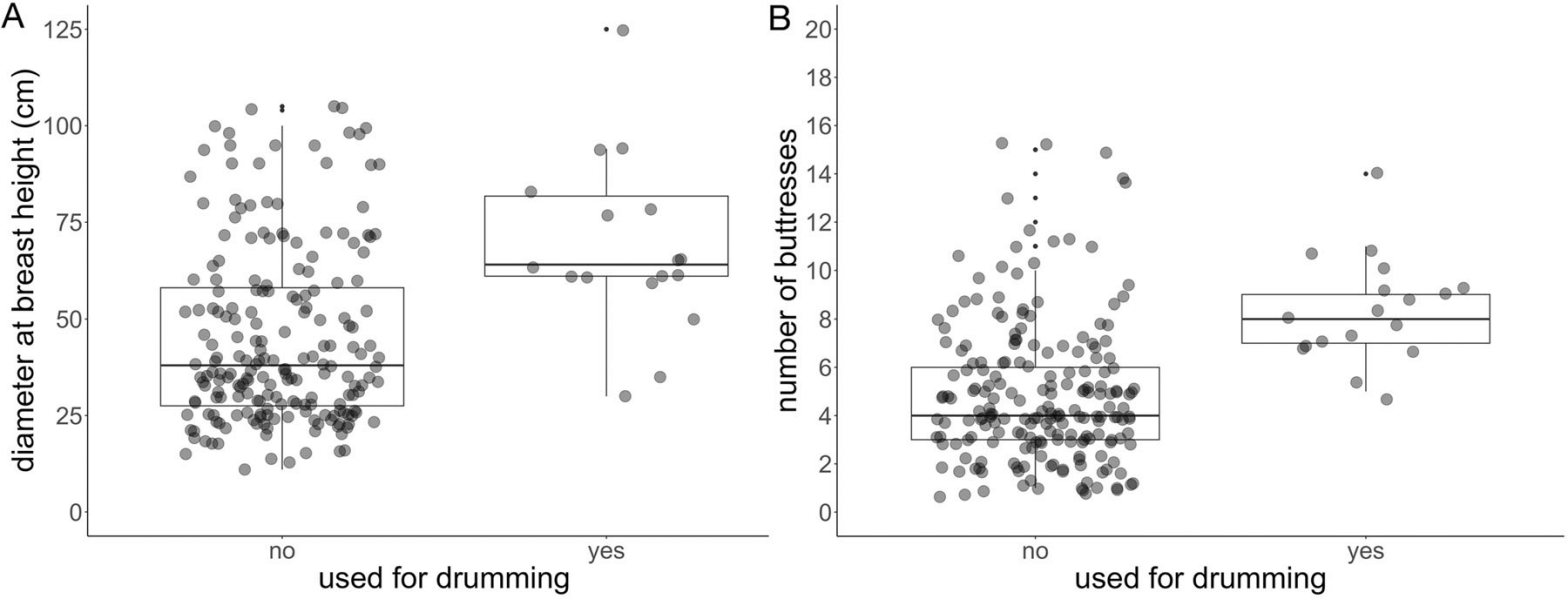
Boxplots showing the Diameter at Breast Height and Number of Buttresses for drumming trees and control trees. (A) Diameter at breast height (cm) for drumming (*N* = 18) and control trees (*N* = 203). (B) Number of buttresses for drumming and control trees.

The final tree selection model included all 221 trees (*N*_drumming_ trees = 18, *N*_control trees_ = 203). The full tree selection model (55 drumming bouts, *N*_observations_ = 221 trees from 42 plots, 32 tree species, and 12 individuals) predicted the use of trees significantly better than the null model (*χ*² = 19.6, *df* = 2, *p* < 0.001; Conditional *R* ^2^ = 0.877; Table 4). Diameter at Breast Height was a clear predictor of tree selection (χ² = 7.82, *p* = 0.005; see Figure 3A), with the exponentiated fixed effect showing an odds ratio of 3.86 (95% CI = 1.50–9.94). With every 1 SD increase of the diameter at breast height the chance of a tree being selected for drumming increased by 286%. Number of Buttresses did not affect the chance of a tree being selected for drumming (*χ*² = 3.0, *p* = 0.08; see Figure 3B), with an odds ratio of 2.58 (95% CI = 0.89–7.49). VIF values indicated there was no collinearity between the fixed effects (VIF_n_buttresses_ = 1.01, VIF_DBH_ = 1.00, VIF_context_ = 1.00). There was no problematic deviation from model assumptions (for model assumption tests, see Supporting Information S1).

**Table 4:** Results of the binomial GLMM exploring the effects of diameter at breast height, and number of buttresses, on the chance of a tree being selected for drumming. Diameter at breast height and number of buttresses were *z*‐transformed before entering the model. Context was included as a control. The table shows estimates, standard errors, test results, and degrees of freedom of the predictors.

	Estimate	SE	*z*	*χ*2	*df*	*p*
(Intercept)	−6.955	3.391	−2.051			(1)
**DBH.z**	**1.350**	**0.483**	**2.796**	**7.815**	**1**	0.005
N_buttresses.z	0.947	0.544	1.740	3.027	1	0.082

*Note:* χ2 and *p*‐values are taken from Wald's Type II chi‐square test. Significant predictors are given in bold. (1) Not indicated because of limited interpretation.

## 4 Buttress Selection

**Figure 4:**
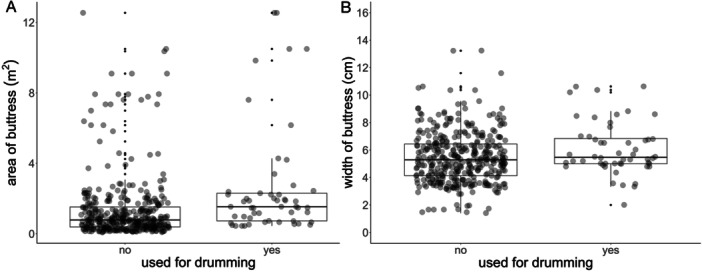
Boxplots showing the area and width of drumming buttresses and control buttresses. (A) Area (m^2^) for drumming buttresses (*N* = 53) and control buttresses (*N* = 380). (B) Width (cm) for drumming and control buttresses.

The final buttress selection model included 433 buttresses (*N*_drumming buttresses_ = 53, *N*_control buttresses_ = 380). The full buttress selection model (52 drumming bouts, *N*_observations_ = 433 buttresses from 52 drumming bouts, 39 trees, 12 individuals, and 6 tree species) predicted the use of buttresses significantly better than the null model (*χ*² = 19.1, *df* = 3, *p* < 0.001; Table 5). Surface area was a significant predictor (*χ*² = 10.6, *p* = 0.001), with the exponentiated fixed effect showing an odds ratio of 1.47 (95% CI = 1.17–1.84). With every 1 SD increase in the surface area, the chance of buttresses being selected for drumming increased by 47% (Figure 4A). Width was also a clear predictor (*χ*² = 6.0, *p* = 0.014), with an odds ratio of 1.40 (95% CI = 1.07–1.84), so a 40% increase in the chance of a buttress being selected with every 1 SD increase of width (Figure 4B). VIF values indicated there was no collinearity between the fixed effects (VIF_area_ = 1.09, VIF_width_ = 1.10, VIF_context_ = 1.11). There was no problematic deviation from model assumptions (for model assumption tests, see Supporting Information S1).

**Table 5:** Results of the binomial GLMM exploring the effects of Buttress Area, and Buttress Width on the chance of a tree being selected for drumming. Area and Width were *z*‐transformed before entering the model. Context was included as a control. The table shows estimates, standard errors, test results, and degrees of freedom of the predictors.

Predictor	Estimate	SE	*z*	*χ* ^2^	*df*	*p*
(Intercept)	−2.360	0.284	−8.300			(1)
**area.z**	**0.387**	**0.119**	**3.255**	**10.597**	**1**	0.001
**width.z**	**0.338**	**0.138**	**2.447**	**5.990**	**1**	0.014

*Note: χ* ^2^ and *p*‐values are taken from Wald's Type II chi‐square test. Significant predictors are given in bold. (1) Not indicated because of limited interpretation.

## 5 Discussion

We found that chimpanzees show selectivity in their choice of drumming buttresses, both in terms of tree species and the physical properties of trees and buttresses. Waibira chimpanzees preferred to drum on two tree species, *Cynometra alexandrii* and *Chrysophyllum albidum*, which were used more often than would be expected given local prevalence. They also preferentially chose larger trees, as well as larger and thicker buttresses.

*C. alexandrii* nor *C. albidum* are not similarly distinctive in terms of the availability or form of their buttresses; instead, each may offer different potential opportunities (for a detailed description of the variation in buttress structure per species, see Supporting Information S3). *C. alexandrii* has a particularly large spread in the buttress area, so while it did not appear to have larger buttresses than other tree species on average, it did have many of the largest buttresses we located during the study—a preferred feature at the buttress level in both the Waibira chimpanzees in Budongo and the Seringbara chimpanzees in Nimba. In contrast, *C. albidum* typically shows a slightly larger number of buttresses than the other drumming tree species. Waibira chimpanzees did not show a clear preference for the number of buttresses, while this was a preference for chimpanzees in Nimba (Fitzgerald et al. 2022). One marked point of contrast between the choices made by Waibira and Seringbara chimpanzees was in buttress width: Fitzgerald et al. (2022) found that the chance of selection increased with decreasing buttress width, whereas we found that chance of selection increased with increasing buttress width.

Importantly tree size and the number of buttresses were strongly correlated, so it is difficult to fully disentangle which factor is shaping chimpanzee drumming tree choice. For example, employing separate models of tree and then buttress selection suggests that chimpanzees first choose a tree, and then—from that tree—choose a buttress. In contrast, chimpanzees may make a direct choice of buttress, ignoring any potential choice at the level of the tree. If so, then the preference for larger trees may simply be a feature of a preference for larger buttresses or for trees with more buttresses. Other features may also impact the likelihood of a tree species being drummed on, for example, chimpanzees sometimes pant‐hoot and drum before climbing a feeding tree and in the morning and evening hours (Clark and Wrangham 1993; Wrangham 1977). Seven of the eight species identified as drumming trees in our study (with the exception of *Bersama abyssinica*) are also a source of food for Waibira chimpanzees, and both *Cynometra* and *Celtis* species are regularly used for nesting (C.H., personal communication).

As chimpanzees appear to attend to tree resonance (e.g., Kalan et al. 2019), a potential explanation of tree species and buttress preferences is that the chosen buttresses offer greater resonance, increasing the travel distance of the sound. Selection based on resonance could explain a preference for larger and thinner buttresses, as found in the Seringbara chimpanzees (Fitzgerald et al. 2022), as the combination of increased surface area and decreased surface thickness improves sound amplification (Yamaha Corporation 2020). But, in contrast, we found a preference in Waibira for thicker buttresses. It is possible that there is a threshold effect, such that a thinner buttress only becomes important once a certain area or width is reached. The comparatively much smaller size of buttresses in Budongo forest as compared to the Seringbara region of Nimba could mean that any threshold is rarely reached in Budongo (Budongo: *M* = 1.43 m^2^, SD = 1.78; Seringbara: 5.63 m^2^, SD = 13.58). Alternatively, it is possible that the lower chimpanzee population density in Seringbara means that it is more important for sound to travel further, while the high population density and presence of other nearby communities in Waibira could make it important to ensure the drumming sound is only heard by nearby parties from within their own community. There is widespread support for the idea that chimpanzees consider both proximate (Schel et al. 2013; Slocombe and Zuberbühler 2007; Soldati et al. 2022) and out‐of‐sight audiences (Hobaiter and Byrne 2012; Hobaiter, Byrne, and Zuberbühler 2017) in their communication. However, there is relatively little work on the interaction between landscape and long‐distance communication, although it has been suggested that they may incorporate elements of terrain for strategic use of their long‐distance vocalizations (Lemoine et al. 2023).

Given the striking variation in the availability of very large, buttressed trees—which were rare in Budongo as compared to Nimba—an interesting question is the extent to which chimpanzees might weigh the trade‐offs between additional travel costs of deviating towards a preferred tree, against the potential benefits of enhanced signal production. One possible means to assess this would be to evaluate the density of preferred drumming trees along well‐established chimpanzee trails (for a similar approach assessing the distribution of food resources via forest elephant trails, see Blake and Inkamba‐Nkulu 2004). If preferences are shared across individuals within a community, we would predict that, in areas of low buttressed‐tree density, trails might have formed to incorporate access to preferred trees during daily travel.

Resonance is affected by multiple factors, one of which is wood density. Tropical hardwood is characterized by low dampening, meaning that the sound is not muffled by the wood itself and may even be amplified (Brémaud et al. 2008). This effect of density is reflected in the preference for hardwoods in the creation of musical instruments (Brémaud et al. 2008; Wegst 2006). *C. alexandrii* has the highest density of any species in our data set, and is considered a tropical hardwood (Mugabi, Banana, and Eikenes 2005); however, *C. albidum* is typically considered a softwood (Reyes et al. 1992). This difference could indicate that wood density is not a primary selective feature for chimpanzees when drumming; alternatively, variation in choice may also reflect variation in function (e.g., short or long‐distance communication) and individual preference (as seen in individual variation in drumming styles, Eleuteri et al. 2022). More substantial datasets at the level of individual chimpanzees and specific contexts are needed to resolve these questions.

It is also possible that the acoustic properties of the wood or buttress form are a secondary consideration when it comes to drumming surface selection. The physical properties of trees and buttresses could increase ease or control over drumming production, allowing individuals to drum more effectively or encode information in drumming structure or rhythm. Chimpanzees employ a variety of techniques for drumming, using both hands and feet, and in some cases drumming from a static position (Eleuteri et al. 2022), whereas in others, drumming is part of a dynamic locomotor display (Arcadi and Wallauer 2013). Drumming may be accompanied at times by the species‐typical long‐distance pant‐hoot vocalization or by other calls—which have their own, additional, acoustic structure. Technique selection may be a feature of the immediate behavioral context or of individual preference; but is also likely shaped by buttress form. Individual chimpanzees show well‐established variation in individual “signature” styles (Arcadi, Robert, and Boesch 1998; Babiszewska et al. 2015; Eleuteri et al. 2022), and it would be particularly interesting to explore whether variation in particular styles is associated with individual preferences in drumming tree species. Future research into the association between physical tree properties and structural or rhythmic drumming bout properties could provide a better understanding of any potential connection between tree preference, drumming technique, and drumming rhythm. However, substantial datasets would be needed to disentangle whether the rhythmic variation is a biproduct of preference for buttress shape and size, or whether preferences for buttresses are driven by the motivation to produce a particular rhythm. Finally, we have considered the 2‐dimensional structural properties of the buttresses drummed on, but chimpanzees may also consider the structural and acoustic properties of the tree as a 3‐dimensional structure. For example, sound propagation is also impacted by reflection from local surfaces—as in the use of resonant surfaces to modify vocalizations in frog and insect species (Lardner and bin Lakim 2002; Tan et al. 2014; Mhatre et al. 2017). Buttress roots that run in close parallel with each other, or in other orientations, may offer opportunities to further amplify or adjust drumming acoustics. Future analyses of the acoustic properties of buttressed trees might allow us to better disentangle these different possible effects.

In summary, eastern chimpanzees of the Waibira community show clear preferences when selecting surfaces for drumming. They drum preferentially on the buttressed trees *C. alexandrii*, a hardwood, and *C. albidum*, a softwood. The chance of a tree being chosen for drumming increases with its diameter, and the chance of a buttress being chosen increases with its surface area and width. Across populations and subspecies, chimpanzees' choice of drumming surface suggests that they are sensitive to the ways in which material properties and form impact the acoustic and structural properties of their drumming signals. We suggest that further attention to the interaction between the physical form of the buttresses and drumming technique, as well as to the ways in which they exploit the acoustic soundscape of trees as a whole, will offer a richer understanding of the ways in which chimpanzees encode information in buttress drumming.

## Author Contributions

**Wytse Wilhelm:** conceptualization (equal), data curation (lead), formal analysis (lead), funding acquisition (supporting), investigation (lead), methodology (equal), visualization (lead), writing–original draft (lead), writing–review and editing (equal). **Vesta Eleuteri:** conceptualization (equal), methodology (equal), writing–review and editing (equal). **Kathelijne Koops:** methodology (equal), writing–review and editing (equal). **Maegan Fitzgerald:** methodology (equal), writing–review and editing (equal). **Klaus Zuberbühler:** resources (equal), writing–review and editing (equal). **Catherine Hobaiter:** conceptualization (equal), data curation (supporting), formal analysis (supporting), funding acquisition (lead), investigation (supporting), methodology (equal), resources (lead), supervision (lead), writing–original draft (equal), writing–review and editing (equal).

## Conflicts of Interest

The authors declare no conflicts of interest.

## Supporting information

Supporting information.

## Data Availability

All data and code are available in a public repository: https://github.com/Wild-Minds/Chimp-Drumming.
