# Supplementary material for: Selectivity in Buttress Drumming Tree Properties Among Chimpanzees (Pan troglodytes schweinfurthii) of the Waibira Community in Budongo Forest, Uganda
Source: Am J Primatol. 2024 Dec 25;87(1):e23712. doi: 10.1002/ajp.23712 (PMC11669765; doi:10.1002/ajp.23712)
Supplement: Supplementary file 1 — Supporting information. [file AJP-87-e23712-s001.pdf]

S1: Testing Assumptions

Figure E1: Model misspecification problems in the tree selection model

To test for model misspecification issues in the tree selection model we used the 'DHARMa' package (Hartig, 2022). The resulting QQ plot (figure E1a) indicated that the residuals were normally distributed (KS test; p=0.49), that there was no over- or under-dispersion in the tree model (Dispersion test; p=0.58) and that there were no significant outliers (Outlier test; p=1). The Residuals vs. Predicted plot (figure E1b) showed slight deviation from the expected values at high residual values, indicating heteroscedasticity in the data. As the combined quantile test was not significant (p=0.06) this was considered to be minor.

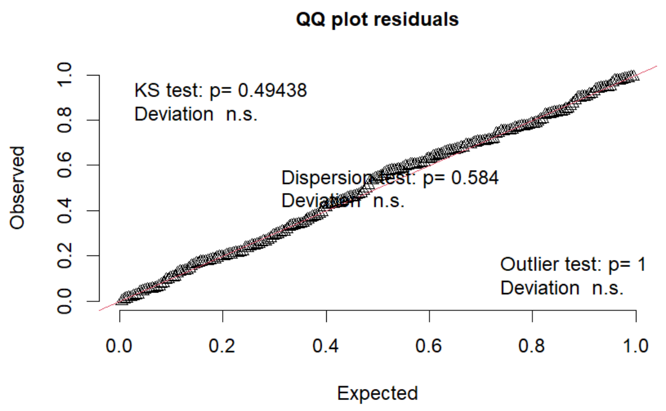

a.

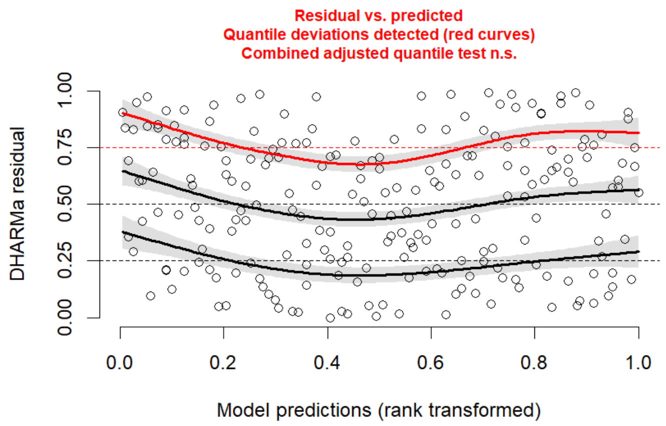

b.

Figure E2: Model misspecification problems in the buttress selection model

To test for model misspecification issues in the buttress selection model we used the 'DHARMa' package (Hartig, 2022). The resulting QQ plot (figure E2a) indicated that the residuals were normally distributed (KS test; p=0.16), that there was no over- or under-dispersion in the tree model (Dispersion test; p=0.94) and that there were no significant outliers (Outlier test; p=1). The Residuals vs. Predicted plot (Figure E2b) indicated the assumptions of linearity and homoscedasticity were met.

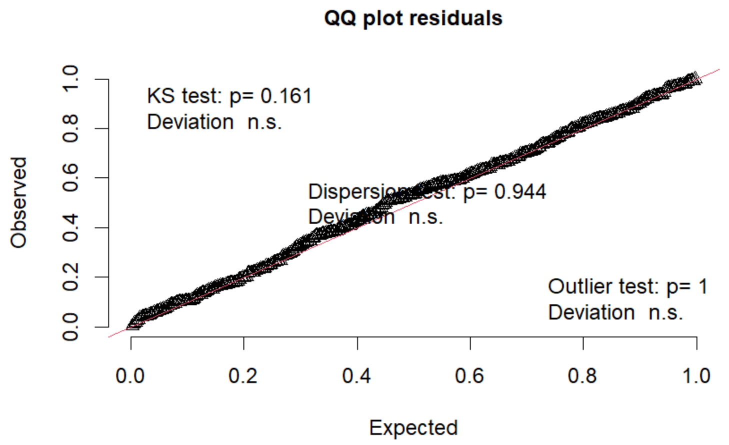

a.

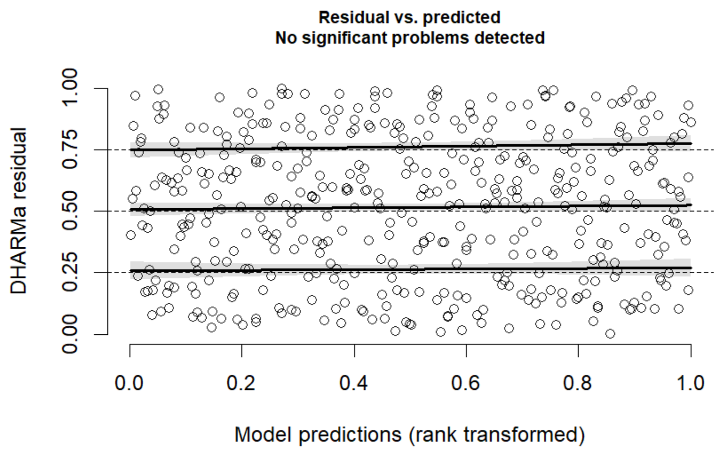

b.

S2: Diameter and Circumference Verification

To test the accuracy of the estimated DBH values (Diameter at Breast Height; available for all trees) we compared the estimated DBH value with a calculated DBH taken from the directly measured CBH (Circumference at Breast Height; available for a subset of trees). This comparison included 157 trees, since only unique trees for which we had values for both the DBH and CBH were included. We compared the variances of each variable using a Levene's Test (Car v3.1-1; Fox & Weisberg, 2019), which showed that the estimated and calculated DBH had equal variances ($p = 0.35$). We performed a Shapiro-Wilk test on each variable, which showed that neither the estimated nor the calculated DBH were normally distributed ($p_{measured} < 0.001$, $p_{calculated} < 0.001$). Because of this, we used the Spearman's Rank Correlation Test for non-parametric data to test the correlation between estimated and calculated DBH. A Spearman's Rank Correlation Test showed the estimated and calculated DBH were highly and significantly correlated ($\rho = 0.94$, $p < 0.001$; Figure 4), suggesting that the estimated DBH provided an accurate assessment of the size of the tree.

Figure E3: Correlation between estimated and calculated DBH

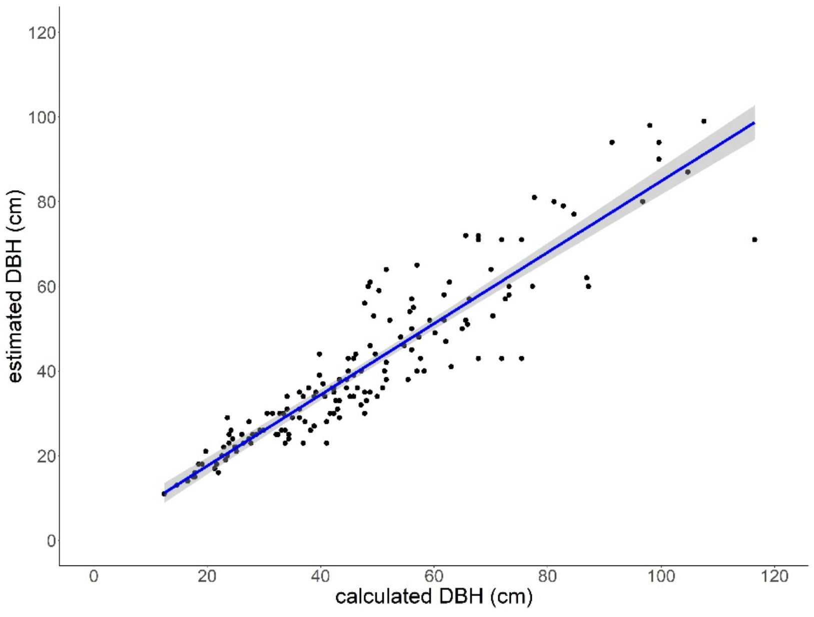

S3: Additional Tables and Figures

Figure E4: Correlation between DBH and Number of Buttresses

We used a Spearman's Ranked Correlation test as DBH was not normally distributed. DBH and Number of Buttresses were positively correlated ($\rho = 0.52$, $p < 0.001$).

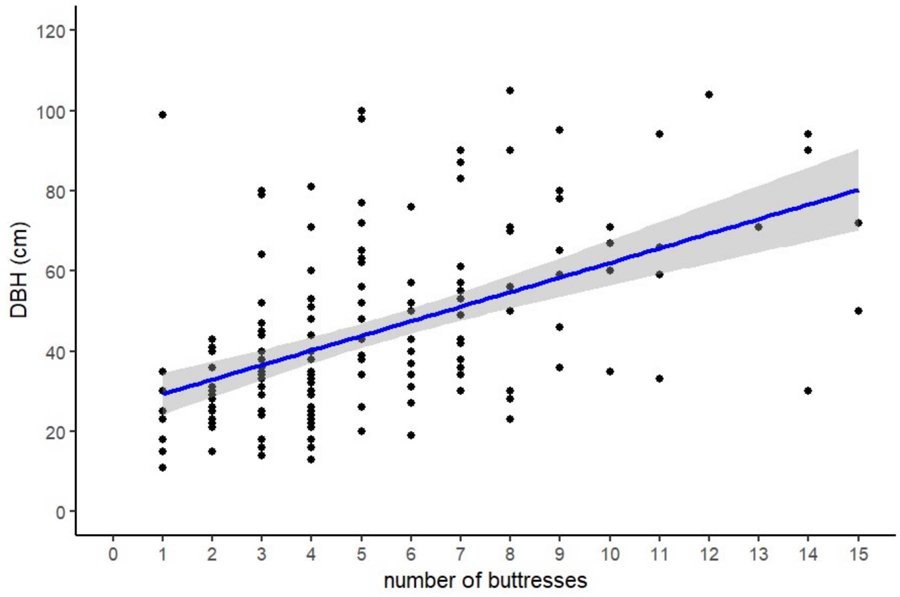

Figure E5: Correlation between DBH and Buttress Area

We used a Spearman's Ranked Correlation test because DBH was not normally distributed. We calculated the average buttress area for each drumming tree; buttress area and DBH were positively correlated ($\rho = 0.67$, $p < 0.001$).

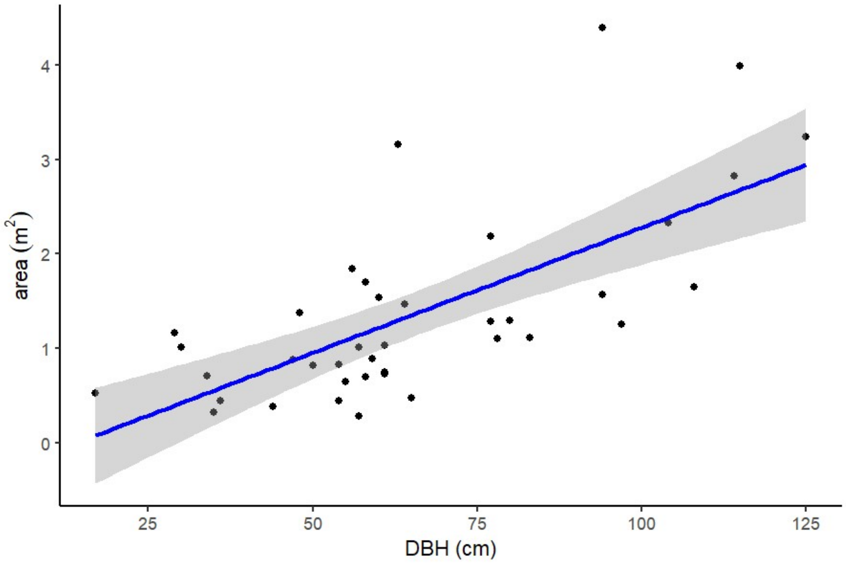

area (m²)
4
3
2
1
0
25
50
75
100
125
DBH (cm)

Table E1: DBH per drumming species

We calculated the average DBH (cm) of the different drumming tree species, as well as for all tree species together. For the last row we included both non-drumming and drumming tree species. For *B. abyssicum* and *F. exasperata* we were only able to locate a single tree, so we do not give the quartiles and standard deviation. The two preferred species (*C. albidum* and *C. alexandrii*) are shown in bold.

Species: *Bersama abyssinica* (BSA); *Chrysophyllum albidum* (CAL); *Celtis mildbraedii* (CMI); *Chrysophyllum muerense* (CMU); *Cynometra alexandrii* (CYA); *Celtis zenkeri* (CZE); *Ficus exasperata* (FA); *Ficus sur* (FSU)

Species	N	Median	1Q	3Q	Mean	SD
BSA	1	43.0			43.0	
**CAL**	**17**	**50.0**	**35.0**	**57.0**	**47.7**	**16.2**
CMI	34	38.0	30.0	51.5	43.1	20.0
CMU	5	77.0	70.0	80.0	74.6	8.1
**CYA**	**53**	**57.0**	**33.0**	**77.0**	**56.5**	**28.9**
CZE	19	25.0	19.0	33.0	27.4	12.4
FE	1	36.0			36.0	
FSU	3	51.0	48.5	67.5	60.3	20.6
All species	197	43.0	29.0	61.0	48.3	24.5

Figure E6: DBH per drumming tree species

The dashed line is the average DBH for all drumming tree species. Preferred species (*C. albidum* and *C. alexandrii*) are shown in red, the others in blue.

Species: *Bersama abyssinica* (BSA); *Chrysophyllum albidum* (CAL); *Celtis mildbraedii* (CMI); *Chrysophyllum muerense* (CMU); *Cynometra alexandrii* (CYA); *Celtis zenkeri* (CZE); *Ficus exasperata* (FA); *Ficus sur* (FSU)

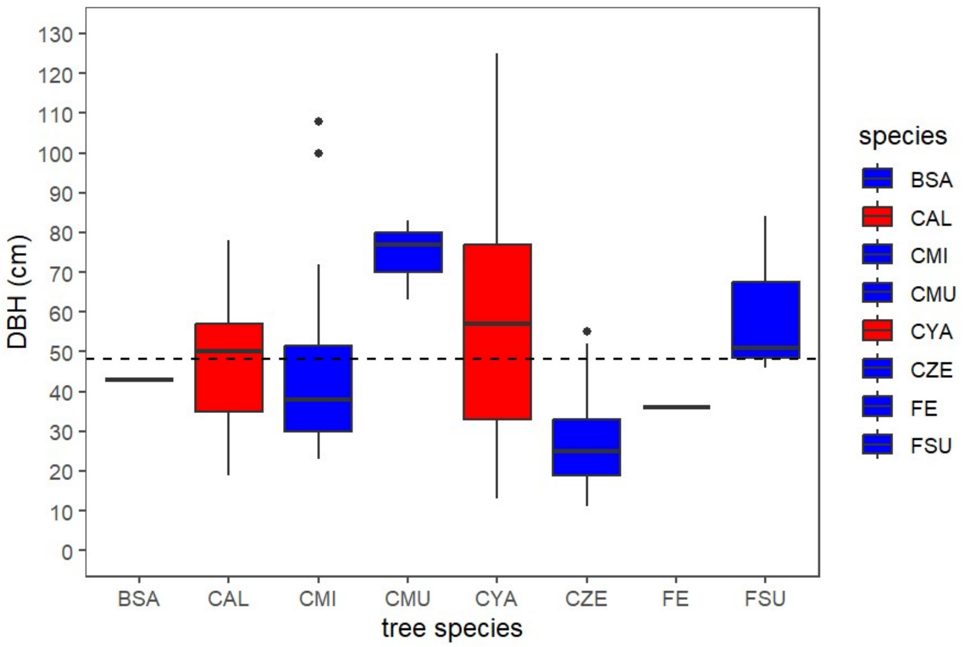

Table E2: Number of Buttresses per drumming species

We calculated the average Number of Buttresses of the different drumming tree species, as well as for all tree species together. For the last row we included both non-drumming and drumming tree species. For *B. abyssicum* and *F. exasperata* we were only able to locate a single tree, so we do not give the quartiles and standard deviation. The two preferred species (*C. albidum* and *C. alexandrii*) are shown in bold.

Species: *Bersama abyssinica* (BSA); *Chrysophyllum albidum* (CAL); *Celtis mildbraedii* (CMI); *Chrysophyllum muerense* (CMU); *Cynometra alexandrii* (CYA); *Celtis zenkeri* (CZE); *Ficus exasperata* (FA); *Ficus sur* (FSU)

Species	N	Median	1Q	3Q	Mean	SD
BSA	1	7			7	
**CAL**	**17**	**8**	**6**	**9**	**7.65**	**2.89**
CMI	34	4.5	3	6	4.56	1.97
CMU	5	7	5	8	6.60	1.52
**CYA**	**53**	**7**	**5**	**10**	**7.57**	**3.63**
CZE	19	4	3	5	4.37	1.98
FE	1	5	5	5	5	
FSE	3	5	4.5	8	6.67	3.79
All species	197	5	3	7	5.72	3.26

Figure E7: Number of Buttresses per drumming species

The dashed line shows the average Number of Buttresses for all drumming species. The two preferred species (*C. albidum* and *C. alexandrii*) are shown in red, the others in blue.

Species: *Bersama abyssinica* (BSA); *Chrysophyllum albidum* (CAL); *Celtis mildbraedii* (CMI); *Chrysophyllum muerense* (CMU); *Cynometra alexandrii* (CYA); *Celtis zenkeri* (CZE); *Ficus exasperata* (FA); *Ficus sur* (FSU)

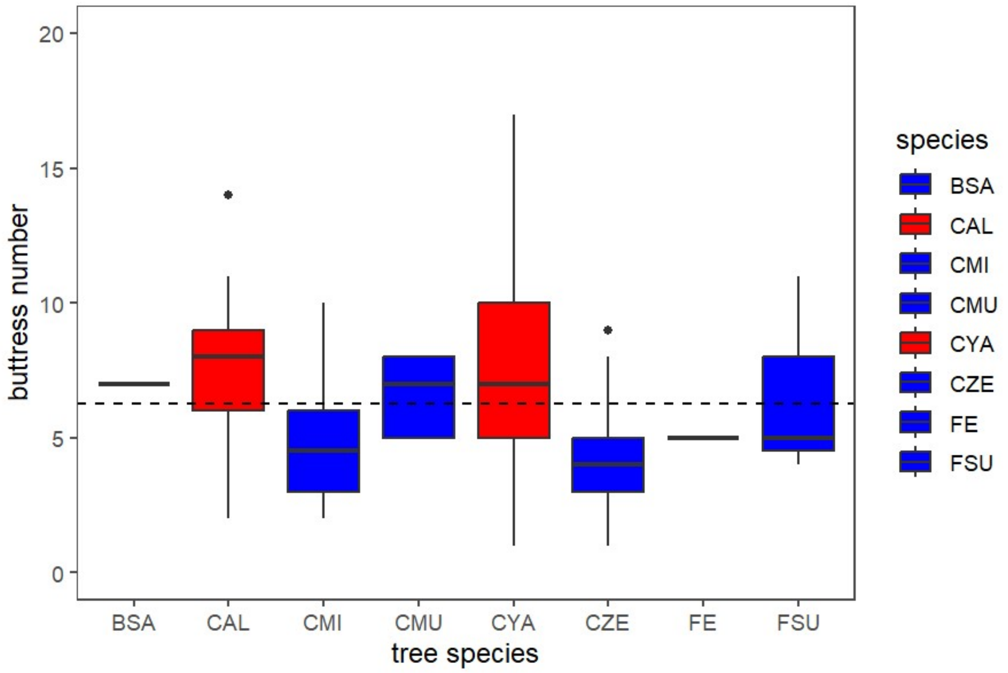

Table E3: Buttress Area per drumming species

We calculated the average Buttress Area (m^2) of the different drumming tree species, as well as for all tree species together. For the last row both non-drumming and drumming tree species are included. The preferred species (*C. albidum* and *C. alexandrii*) are shown in bold.

Species: *Chrysophyllum albidum* (CAL); *Celtis mildbraedii* (CMI); *Chrysophyllum muerense* (CMU); *Cynometra alexandrii* (CYA); *Celtis zenkeri* (CZE); *Ficus exasperata* (FA)

Species	N	Median	1Q	3Q	Mean	SD
**CAL**	**87**	**0.72**	**0.32**	**1.34**	**0.98**	**0.86**
CMI	36	0.81	0.49	1.78	1.12	0.93
CMU	10	2.02	1.83	2.81	2.67	2.09
**CYA**	**171**	**0.98**	**0.45**	**2.00**	**1.73**	**2.20**
CZE	16	0.60	0.41	1.0	0.99	0.59
FE	5	0.39	0.37	0.51	0.45	0.14
All species	325	0.88	0.42	1.78	1.43	1.78

Figure E8: Buttress Area (M^2) per drumming species

The dashed line shows the average Buttress Area for all drumming tree species. The preferred species (*C. albidum* and *C. alexandrii*) are shown in red, the others in blue.

Species: *Chrysophyllum albidum* (CAL); *Celtis mildbraedii* (CMI); *Chrysophyllum muerense* (CMU); *Cynometra alexandrii* (CYA); *Celtis zenkeri* (CZE); *Ficus exasperata* (FA)

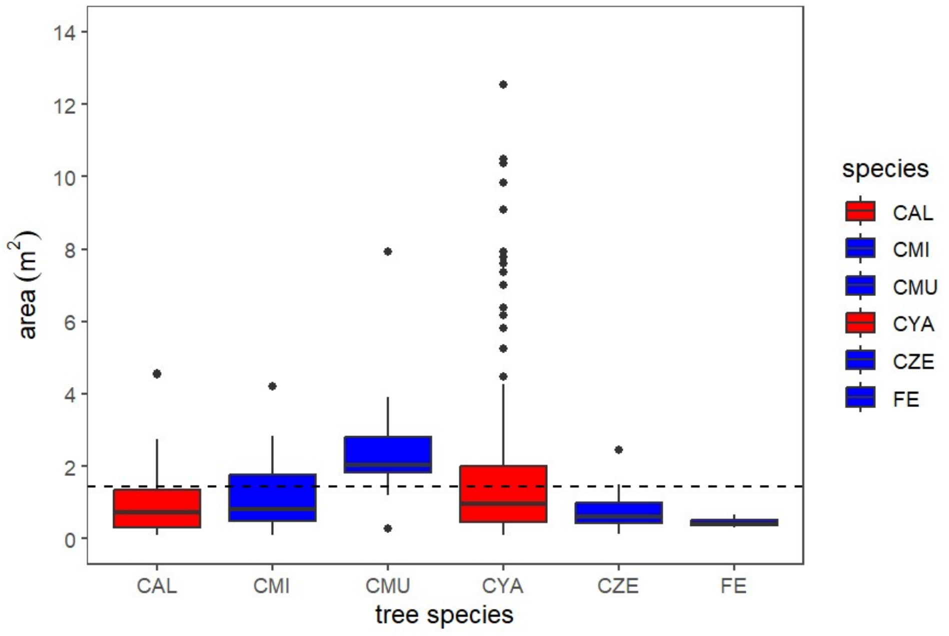

Table E4: Buttress Width per drumming species

We calculated the average Buttress Width (cm) of the different drumming tree species, as well as for all tree species together. For the last row both non-drumming and drumming tree species are included. The preferred species (*C. albidum* and *C. alexandrii*) are shown in bold.

Species: *Chrysophyllum albidum* (CAL); *Celtis mildbraedii* (CMI); *Chrysophyllum muerense* (CMU); *Cynometra alexandrii* (CYA); *Celtis zenkeri* (CZE); *Ficus exasperata* (FA)

Species	N	Median	1Q	3Q	Mean	SD
**CAL**	**87**	**5.90**	**4.72**	**7.05**	**6.01**	**1.87**
CMI	36	5.63	5.06	6.64	6.22	2.71
CMU	10	7.72	5.70	8.64	7.65	2.28
**CYA**	**171**	**4.83**	**3.62**	**6.02**	**4.99**	**1.78**
CZE	16	4.47	3.98	5.81	4.82	1.09
FE	5	7.80	7.33	8.60	8.24	1.22
All species	325	5.27	4.20	6.67	5.53	2.04

Figure E9: Buttress Width per drumming species

The dashed line shows the average Buttress Width (cm) for all drumming tree species. The preferred species (*C. albidum* and *C. alexandrii*) are shown in red, the others in blue.

Species: *Chrysophyllum albidum* (CAL); *Celtis mildbraedii* (CMI); *Chrysophyllum muerense* (CMU); *Cynometra alexandrii* (CYA); *Celtis zenkeri* (CZE); *Ficus exasperata* (FA)

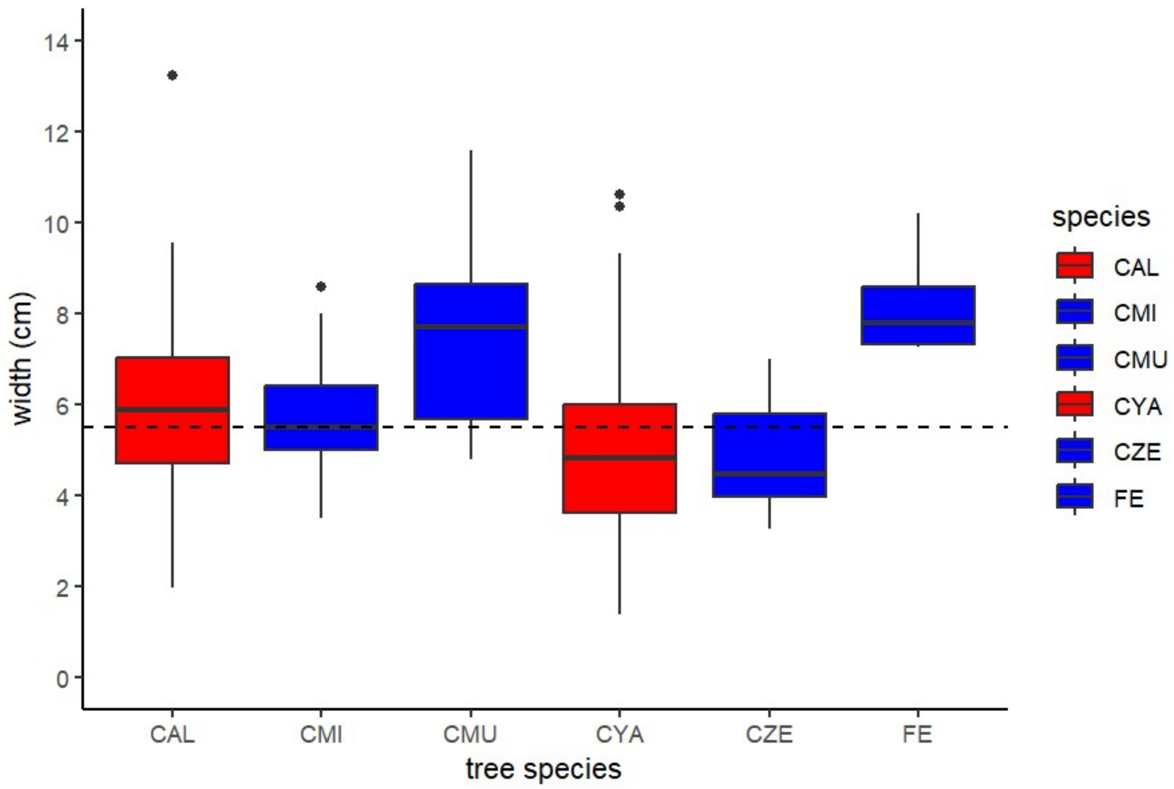

Figure E10: Tree species used by context

The tree species proportions used in the different drumming contexts (Display and Other). Species: *Bersama abyssinica* (BSA); *Chrysophyllum albidum* (CAL); *Celtis mildbraedii* (CMI); *Chrysophyllum muerense* (CMU); *Cynometra alexandrii* (CYA); *Celtis*

zenkeri (CZE); *Ficus exasperata* (FA); *Ficus sur* (FSU)

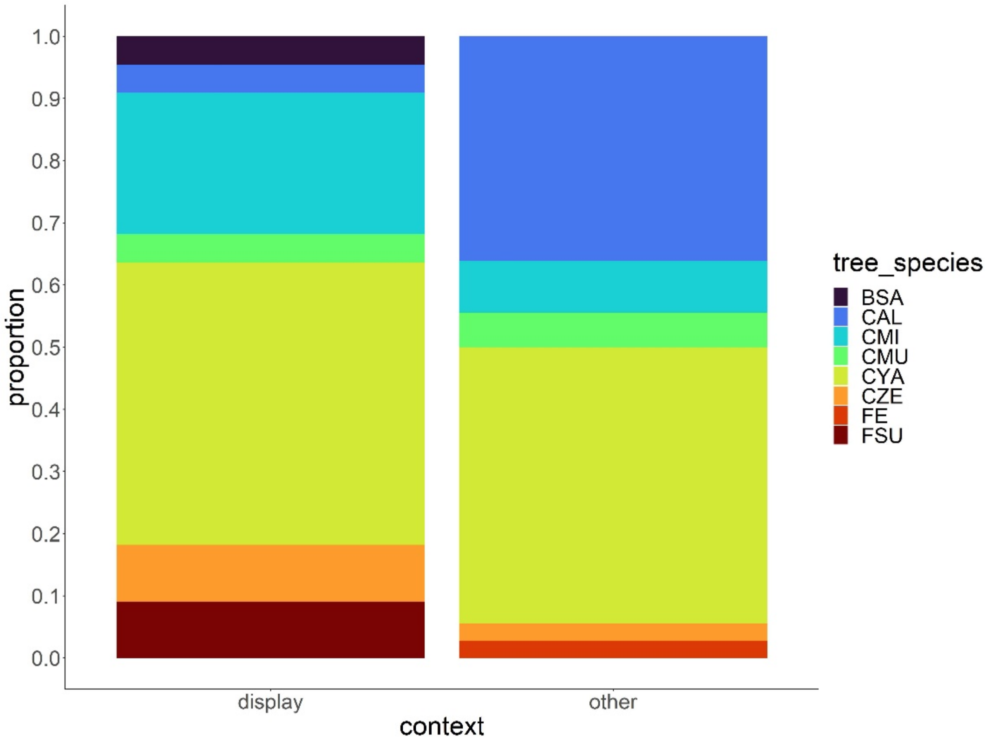

S4: Additional GLMMs

Table E5: Three-way interaction tree selection model

We ran a binomial GLMM exploring the effects of a three-way interaction between DBH, Number of Buttresses, and Context on the chance of a tree being selected for drumming. We z-transformed DBH and Number of Buttresses before entering them into model. (1) Not indicated because of limited interpretation.

Predictor	Estimate	SE	z	χ^2	df	p
(Intercept)	-10.794	7.231	-1.493			(1)
DBH.z	5.742	4.095	1.402	0.630	1	0.427
N_buttresses.z	1.439	2.811	0.512	0.370	1	0.543
Context_other	1.064	3.945	0.270	0.691	1	0.406
DBH.z : N_buttresses.z	-1.074	2.354	-0.456	1.297	1	0.255
DBH.z : Context_other	-3.988	4.002	-0.997	1.841	1	0.175
N_buttresses.z : Context_other	1.710	2.856	0.599	0.694	1	0.405
DBH.z : N_buttresses.z : Context_other	-0.241	2.499	-0.096	0.009	1	0.923

Table E6: Double interaction tree selection model

We ran a binomial GLMM expiring the effects of interactions between DBH and Context and Number of Buttresses and Context on the chance of a tree being selected for drumming. We z-transformed DBH and Number of Buttresses before entering them into model. (1) Not indicated because of limited interpretation.

Predictor	Estimate	SE	z	χ^2	df	p
(Intercept)	-8.208	4.778	-1.718			(1)
DBH.z	4.014	2.524	1.590	1.641	1	0.200
N_buttresses.z	0.134	1.155	0.116	0.618	1	0.432
Context_other	0.515	2.389	0.216	0.852	1	0.356
DBH.z : Context_other	-3.641	2.822	-1.290	1.665	1	0.197
N_buttresses.z : Context_other	2.143	1.840	1.165	1.357	1	0.244

Table E7: Single interaction tree selection modelWe ran a binomial GLMM exploring the effects of an interaction between DBH and Number of Buttresses and the main effect Context on the chance of a tree being selected for drumming. We z-transformed DBH and Number of Buttresses before entering them into the model. (1) Not indicated because of limited interpretation.

Predictor	Estimate	SE	z	χ^2	df	p
(Intercept)	-9.832	6.708	-1.466			(1)
DBH.z	1.995	1.463	1.363	0.335	1	0.563
N_buttresses.z	2.716	1.604	1.694	2.213	1	0.137
Context_other	-1.819	1.154	-1.576	2.485	1	0.115
DBH.z : N_buttresses.z	-1.147	0.929	-1.236	1.527	1	0.217

Table E8: Drummer identity as random effectWe ran a chi-square analysis of variance between the tree selection model without drummer identity (Ind_ID) included as random effect, and the tree selection model with drummer identity included as random effect (χ^2 = 0.00, p = 0.99).

	Npar	AIC	BIC	logLik	Deviance	χ^2	df	p
Without Ind_ID	6	95.836	116.22	-41.918	83.836			
With Ind_ID	7	97.836	121.62	-41.918	83.836	0.000	1	0.991

Table E9: Three-way interaction buttress selection modelWe ran a binomial GLMM exploring the effects of a three-way interaction between Buttress Area, Buttress Width, and

Context on the chance of a buttress being selected for drumming. We z-transformed Area and Width before entering them into model. (1) Not indicated because of limited interpretation.

Predictor	Estimate	SE	z	χ^2	df	p
(Intercept)	-2.290	0.297	-7.701			(1)
area.z	0.293	0.209	1.353	10.144	1	0.001
width.z	0.173	0.273	0.632	5.823	1	0.016
Context_other	0.362	0.352	1.028	1.716	1	0.190
area.z : width.z	0.102	0.134	0.759	0.044	1	0.833
area.z : Context_other	0.015	0.293	0.050	0.072	1	0.788
width.z : Context_other	0.192	0.336	0.572	0.824	1	0.774
area.z : width.z : Context_other	-0.543	0.360	-1.511	2.282	1	0.131

Table E10: Double interaction buttress selection model

We ran a binomial GLMM exploring the effects of double interactions between Buttress Area and Context, and Buttress Width and Context on the chance of a buttress being selected for drumming. We z-transformed Area and Width before entering them into the model. (1) Not indicated because of limited interpretation.

Predictor	Estimate	SE	z	χ^2	df	p
(Intercept)	-2.337	0.299	-7.808			(1)
area.z	0.377	0.159	2.365	10.667	1	0.001
width.z	0.315	0.202	1.554	6.058	1	0.014
Context_other	0.405	0.353	1.145	1.656	1	0.198
area.z : Context_other	0.029	0.241	0.122	0.015	1	0.903
width.z : Context_other	0.048	0.277	0.173	0.030	1	0.863

Table E11: Single interaction buttress selection modelWe ran a binomial GLMM exploring the effects of an interaction between Buttress Area and Buttress Width, and the main effect Context, on the chance of a buttress being selected for drumming. We z-transformed Area and Width before entering them into model. (1) Not indicated because of limited interpretation.

Predictor	Estimate	SE	z	χ^2	df	p
(Intercept)	-2.360	0.285	-8.287			
area.z	0.387	0.127	3.036	10.600	1	0.001
width.z	0.338	0.153	2.210	5.990	1	0.014
Context_other	0.430	0.337	1.277	1.630	1	0.202
area.z : width.z	-0.000	0.088	-0.004	0.000	1	0.997

S5: Direct Replication of Fitzgerald et al. (2022)

To allow for direct comparison with the results of Fitzgerald et al. (2022) we ran additional analyses on our data using the exact same methods and R code (Fitzgerald et al., 2022, supplementary materials). This meant that the predictors were not z-transformed, and that context was not included as a predictor.

Tree selection model

The binomial GLMM for the tree selection included 221 observations from 42 plots. We included only Drumming Tree ID (equivalent to Plot ID in Fitzgerald et al. (2022) as a random effect (our main models also included Individual and Tree Species as random effects). A χ^2 likelihood ratio test showed the full model predicted tree selection better than the null model (χ^2=35.6, p<0.001). The conditional R^2 was 0.426. The odds of a tree being selected for drumming increased with DBH (OR=1.03, 95% CI=1.01-1.06, p=0.012) and with number of buttresses (OR=1.32, 95% CI=1.06-1.64, p=0.015). This meant that the odds of a tree being selected for drumming increased with about 3% with each additional cm DBH, and with about 32% for each additional buttress. VIF values indicated no collinearity between the predictors (VIF_{DBH}=1.01, $VIF_{n\_buttresses}$=1.01).

Table E12: Tree selection replication model

The table shows the results of the replicated model on tree selection. Significant predictors are given in bold. [(1)] Not indicated because of limited interpretation.

Predictor	Estimate	SE	z	χ^2	df	p
(Intercept)	-6.252	1.500	-4.168			(1)
**DBH**	**0.031**	**0.012**	**2.513**	**6.317**	**1**	**0.012**
**N_buttresses**	**0.275**	**0.113**	**2.436**	**5.934**	**1**	**0.015**

Buttress selection

The binomial GLMM for the buttress selection included 433 observations from 17 trees. Unlike Fitzgerald et al. (2022) we did not nest Drumming Tree IDs inside a Plot ID value for the random effects, as our buttress dataset did not include any drumming trees from the same plot (two drumming trees within 10 m of each other). Unlike in the main model Individual, Tree Species, and Drum ID were not included as random effect variables. A χ^2 likelihood ratio test showed that the full model did not predict buttress selection better than the null model (χ^2=0.5, p=0.776). The conditional R^2 was 0.950. Neither area nor width were significant predictors in the full model.

Table E12: Buttress selection replication model

The table shows the results of the replicated model on buttress selection. [(1)] Not indicated because of limited interpretation.

Predictor	Estimate	SE	z	χ^2	df	p
(Intercept)	-9.739	2.942	-3.310			<0.001
Area	-0.090	0.123	-0.733	0.537	1	0.464
Width	0.033	0.167	0.200	0.040	1	0.842
